# Success Comes at a Cost, Even for Phages

**DOI:** 10.1371/journal.pbio.0040227

**Published:** 2006-06-13

**Authors:** Liza Gross

Living is an energy-intensive exercise that inevitably involves trade-offs. As many a mother may tell you, expending the energy necessary to raise a clutch of kids can shave years off one's life. Trade-offs between reproductive success and survival have been demonstrated for a wide variety of organisms and, in keeping with life history theory, should arise in any organism striving to maximize fitness under the constraints of finite resources.

In a new study, Marianne De Paepe and François Taddei asked whether these trade-offs extend to viruses. Though not universally considered alive because they can't replicate without the help of their host's molecular machinery, viruses pass through distinct life cycle stages, mutate, and evolve in response to selection pressures from their host. Viruses also have life history traits, such as multiplication rate in a host, survival outside the host, and mode of transmission.

Working with viruses that infect bacteria, called bacteriophages (or in this case, coliphages, which infect *Escherichia coli*), De Paepe and Taddei predicted that the phage, just like a full-fledged cellular organism, would display trade-offs between survival and reproduction. They discovered that, although coliphages don't wither and die like “real” organisms, they do experience life history trade-offs, with rapid reproducers suffering higher casualties outside the host. And, by investigating several physical properties of the coliphages, they found that two physical parameters account for most of the observed variation in survival.

During infection, phages rapidly reproduce until the bacterial cell ruptures (called lysis) and releases the virions (viral particles, which consist of little more than the viral genome encased in a capsid protein shell). A phage typically meets its end after encountering harsh conditions, such as heat or osmotic shock, that rupture the capsid, releasing its genetic contents. How well the phage can resist such stresses depends in part on the strength of the capsid, which must withstand the extreme pressure exerted by strong repulsive forces between charged DNA strands and bending of the tightly packed DNA.

To investigate the presence of life history trade-offs in phages, De Paepe and Taddei measured the decay rate (mortality) and multiplication rate of 16 coliphage strains. They determined the kinetics of mortality within and across the phage populations, by measuring the number of phage particles that completed an infectious cycle in *E. coli* cultures, at different times and temperatures. Although all the phages died at a constant rate over time, different strains showed considerable variation in these rates. The constant rate (meaning that the probability of death did not increase over time) suggests that the phages succumbed to a single event—indicating that they don't age—rather than to a series of events, which would be consistent with aging. Decay rates increase exponentially with the inverse of the temperature, which is a characteristic of very simple chemical reactions.

To determine the factors underlying this variation, the researchers investigated the correlation between several physical parameters and decay rate. Among the parameters measured were two factors governing capsid stability—density of the packaged genome and “surfacic mass of the capsid,” an indicator of capsid thickness—and two indicators of replication rate—burst size (number of particles released during an infection cycle) and latency period (time between infection and host lysis). They found that decay rate “increases significantly” with the density of the packaged DNA, linking higher internal pressure with higher mortality. By contrast, decay rate decreases as surfacic mass increases, supporting the notion that a stronger capsid increases phage stability. The highest correlation was between decay rate and multiplication rate in the bacterial host. (Multiplication rate was determined by dividing burst size by latency period.) A model based on these three variables—packaged DNA density, surfacic mass, and multiplication rate—accounts for over 90% of the observed variability in phage mortality rates.

Even though they don't have their own metabolism, viruses experience the same sorts of trade-offs between survival and reproduction seen in a wide range of species. This finding suggests that models of virulence evolution, which assume that transmission rates increase along with virulence, may not be valid, since transmission depends not just on parasite multiplication rate but also on survival—which, they show, are negatively correlated. “The fact that this trade-off is present in this very simple biological situation,” the researchers write, “suggests that it might be a fundamental property of evolving entities produced under constraints.” If this is true, the “nonliving” phages that opened the door to some of the most important discoveries in molecular biology may well provide a similar service for a wide range of evolutionary phenomena.

## 

**Figure:**
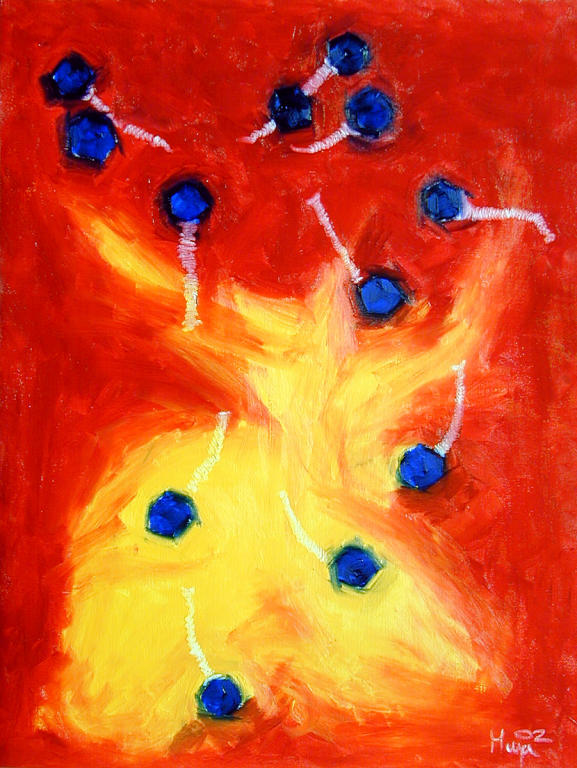
Viruses infecting the bacterium *Escherichia coli* present very different life histories, which are probably constrained by a tradeoff between the decay rate of virus particles and the multiplication rate in the bacterial host. (Painting: “Phagedancer” by Stefanie Timmermann)

